# *J*_*e*_(4.2 K, 31.2 T) beyond 1 kA/mm^2^ of a ~3.2 μm thick, 20 mol% Zr-added MOCVD REBCO coated conductor

**DOI:** 10.1038/s41598-017-06881-x

**Published:** 2017-07-31

**Authors:** A. Xu, Y. Zhang, M. Heydari Gharahcheshmeh, Y. Yao, E. Galstyan, D. Abraimov, F. Kametani, A. Polyanskii, J. Jaroszynski, V. Griffin, G. Majkic, D. C. Larbalestier, V. Selvamanickam

**Affiliations:** 1 Department of Mechanical Engineering, the Texas Center for Superconductivity, and Advanced Manufacturing Institute, University of Houston, Houston, TX 77204 USA; 2 Applied Superconductivity Center, National High Magnet Field Laboratory, Florida State University, Tallahassee, FL 32310 USA

## Abstract

A main challenge that significantly impedes REBa_2_Cu_3_O_x_ (RE = rare earth) coated conductor applications is the low engineering critical current density *J*_*e*_ because of the low superconductor fill factor in a complicated layered structure that is crucial for REBa_2_Cu_3_O_x_ to carry supercurrent. Recently, we have successfully achieved engineering critical current density beyond 2.0 kA/mm^2^ at 4.2 K and 16 T, by growing thick REBa_2_Cu_3_O_x_ layer, from ∼1.0 μm up to ∼3.2 μm, as well as controlling the pinning microstructure. Such high engineering critical current density, the highest value ever observed so far, establishes the essential role of REBa_2_Cu_3_O_x_ coated conductors for very high field magnet applications. We attribute such excellent performance to the dense *c*-axis self-assembled BaZrO_3_ nanorods, the elimination of large misoriented grains, and the suppression of big second phase particles in this ~3.2 μm thick REBa_2_Cu_3_O_x_ film.

## Introduction

Significant opportunities exist for high temperature cuprate superconductors in high field magnets with potential fields well beyond the limit of low temperature superconducting (LTS) technology^1, 2^. In this respect, REBa_2_Cu_3_O_x_ (REBCO, where RE = rare earth) coated conductors stand out owing to the strong mechanical stress tolerance and high current-carrying capability^3^. Recently, REBCO coated conductors are being used in the design and construction of a 32 T all-superconducting magnet (under test), where the REBCO insert magnet produces 17 T in a background 15 T provided by a LTS magnet^4^. REBCO coated conductors have also been demonstrated in a magnet with an overall 42.5 T field generated by a 11.3 T REBCO insert coil in a 31.2 T background field^5^, a 26 T superconducting magnet^6^, and the concept design of a 100 T magnet^7^. Moreover, prototype coils for rotation machinery applications^8^, superconducting magnetic energy storage^9^, magnetic resonance imaging and nuclear magnetic resonance^10–12^, and high energy physics^13, 14^ are being extensively investigated. A critical need for high field magnet R&D is the substantially increased performance and reduced cost, and aggressively pursuing the development of superconducting magnets^15^.

High critical current density, *J*_*c*_ of REBCO coated conductors is pursued through a broad range of nanoscale defects with size comparable to the coherence length, which act as pinning centers to immobilize the magnetic vortices to maintain the dissipation free current^1^. Various nanoparticles, such as RE_2_O_3_ and BaZrO_3_ (BZO), have been well established as effective pinning centers enhancing *J*_*c*_ at high temperatures and recently at low temperatures too^16–21^. More specifically for very high field magnet applications at 4.2 K, BMO (M is metal) nanorods have attracted extensive attention owing to their strong pinning efficiency. At low temperatures *T*, BMO nanorods provide correlated pinning around the *c*-axis, and more importantly introduce the weak point pins substantially raising *J*_*c*_ at all magnetic field directions^22–24^. One important example is the record *J*_*c*_ ~ 8.5 MA/cm^2^ at 4.2 K and 20 T and extremely high pinning force density *F*_*p*_ ∼ 1.7 TN/m^3^ attained by the incorporation of high density of BZO nanorods into the metalorganic chemical vapor deposited (MOCVD) REBCO films via 15 mol% Zr-addition^22^. Such high values are more than three times higher than that of the nominal pure samples and well above any other available superconductors. Recently, BaHfO_3_ (BHO) nanorods have attracted more attention in pulsed laser deposited (PLD) REBCO films. High *F*_*p*_ ∼ 1.6 TN/m^3^, comparable to our 15% Zr-added REBCO films, in a ~0.26 μm thick SmBa_2_Cu_3_O_y_ film, and *F*_*p*_ ~ 1.2 TN/m^3^ in a ~3.3 μm thick EuBa_2_Cu_3_O_y_ film have been achieved, via BaHO nanorod incorporation^23, 25^.

A distinctive feature of REBCO coated conductors is the extremely low superconductor fill factor, 1–2% whereas it is 25–40% for Bi_2_Sr_2_CaCu_2_O_x_ (Bi2212) and (Bi,Pb)_2_Sr_2_Ca_2_Cu_3_O_10_ (Bi2223) and up to 50% for Nb-Ti and Nb_3_Sn^26^. This low fill factor results from the typical layer structure of REBCO coated conductors to sustain highly biaxially texture of REBCO films^27^. The low fill factor has so far limited the engineering critical current density *J*_*e*_ of REBCO coated conductors^26^. Growing thicker REBCO layer is a direct and attractive strategy to increase *J*_*e*_. Theoretically, in the presence of dense strong three dimensional (3D) pins, high and thickness independent *J*_*c*_, regardless of thickness is feasible^28^. RE_2_O_3_ precipitates and BZO nanorods have been demonstrated as strong 3D pins in modern REBCO coated conductors^29, 30^. Unfortunately, these observations have been restricted to REBCO films mostly below 1 μm. Further thickness increase is always accompanied with lower *J*_*c*_, sometimes even with lower *J*_*e*_^31^. This feature is ascribed to the degraded microstructure due to the extremely sensitivity of REBCO performance to the growth conditions. This situation is even worse with increasing Zr-addition, which limits the commercial REBCO coated conductors thickness at about ∼1.5 μm, and Zr addition below ∼10%, and makes the homogeneity a grand challenge for the large scale production of REBCO coated conductors^32^.

Utilizing multi-pass MOCVD process, we have recently successfully grown REBCO layer thickness up to ~3.2 μm and with champion *J*_*e*_ exceeding 1 kA/mm^2^ at 4.2 K and *H* up to 31.2 T. In comparison with the previous ~3 μm thick REBCO films, this recent sample shows substantially reduced current blocking effects and uniformly distributed pinning centers. We attribute such homogeneous microstructure to the significantly reduced deposition time in one pass. Because the tape temperature is not directly monitored in the conventional MOCVD system used in this work, it is very difficult to grow a high quality ~3 μm thick film in one pass. By dividing the long, ~3 μm thick REBCO layer deposition into three short single-pass depositions, we not only shortened the deposition time, but also adjust the process individually in each pass based on the performance of previously-deposited layer. Here, we will concentrate on the *J*_*e*_ enhancement of this ~3.2 μm thick film for high field magnet applications, and the homogeneous microstructure that contributes to this high *J*_*e*_.

## Results

**Figure 1:**
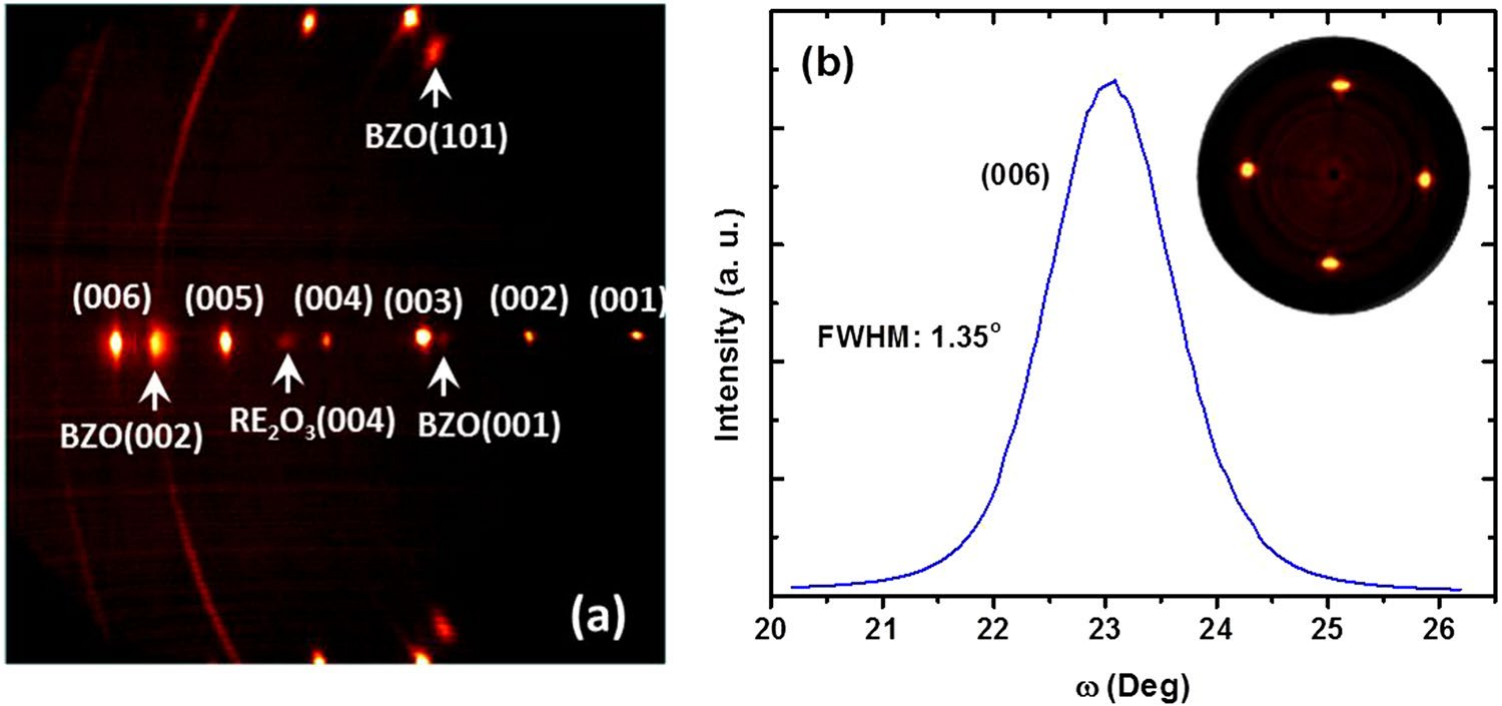
(**a**) GADDS 2D x-ray diffraction patterns from the ∼3.2 μm thick, 20% Zr-added REBCO film at χ = 90° to highlight the high quality crystallinity. (**b**) ω-scan to emphasize the out-of-plane texture. The inset of (**b**) is the pole figure of REBCO (103) peak. The sharp, four-fold symmetric peaks indicate a strong in-plane texture of REBCO matrix.

Figure 1 summarizes the x-ray diffraction (XRD) results. A clearly evident feature of this ~3.2 um thick sample is the high crystalline quality, close to that of the corresponding ~1 μm thick films^33^. In the two-dimensional XRD pattern recorded using a general area-detector diffractometer system (GADDS) shown in Fig. 1(a), the sample shows sharp and strong REBCO peaks, no diffraction rings from REBCO being observed. Besides the evident (00l) peaks, there are distinct BZO (101) and RE_2_O_3_ (004) peaks, resulted from the growth of BZO nanorods and RE_2_O_3_ precipitates inside the REBCO matrix. No other second phases are observed from the GADDS scan. The only observed faint diffraction ring is from the Hastelloy substrate. The detailed texture information is shown in Fig. 1(b). The full width at half maximum (FWHM) of omega scan (Δω) of REBCO (006) peak is 1.35°, comparable to that of ~0.8 μm thick BZO-added pulsed laser deposited YBCO films^34^. The strong and sharp four-fold symmetric peaks of the pole figure for REBCO (103) peak highlight the strong in-plane texture.

**Figure 2:**
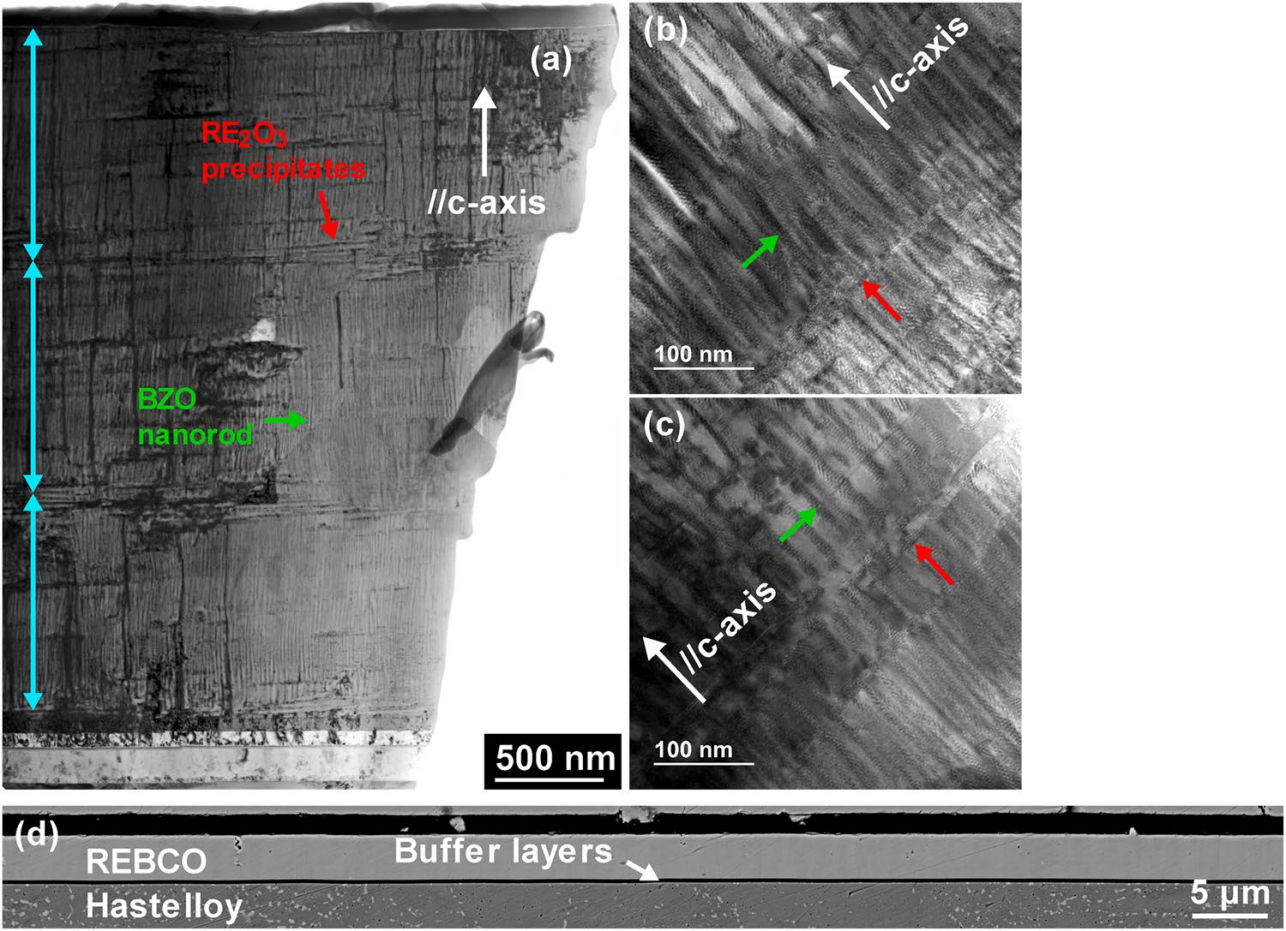
(**a**) Low magnification cross-section TEM image viewed along the current-flowing direction. (**b**) High magnification cross section TEM image of the REBCO film grown in a pass. (**c**) High magnification cross section TEM image of the REBCO layer at the interface between two passes. (**d**) Back-scattered electron image of film cross section to emphasize the homogeneous microstructure and the absence of large misoriented grains and big second phase particles acting as current blocking effects. Occasional small second phase particles with size below 1 μm are observed. All cracks and scratches in (**d**) are from sample polishing.

Figure 2 reveals the homogeneous microstructure of this ~3.2 μm thick film. Figure 2(a) is a low magnification transmission electron microscopy (TEM) image to examine the microstructure features of the entire cross section through the ~3.2 µm thickness. A high density of self-assembled BZO nanorods distribute homogeneously through the thickness along the *c*-axis, except for the interface between different passes, where the in-plane RE_2_O_3_ precipitates interrupt the continuous growth of BZO nanorods. Besides the BZO nanorods, there are also in-plane aligned RE_2_O_3_ precipitate arrays. In comparison to the BZO nanorods, the RE_2_O_3_ precipitate arrays are distributed homogeneously in the microstructure of the films grown within each pass; however, from the substrate to the sample free surface, the density of the RE_2_O_3_ precipitate arrays increases from the first pass, to the second pass, and then the third pass. Besides the BZO nanorods and the RE_2_O_3_ precipitate arrays, there are also sparse threading dislocations along the *c*-axis. We will not consider the pinning effects from the threading dislocations because of their low density. Figure 2(b) and (c) present detailed microstructures of the film grown in one pass and the interface between two passes. Within the film grown in a pass, the BZO nanorods grow continuously and are decoupled occasionally by stacking faults and RE_2_O_3_ precipitate arrays. On the other hand, at the interface zone, a lower density of shorter BZO nanorods, ~100 nm along the thickness direction, are evident. Meanwhile, the densities of stacking faults and RE_2_O_3_ precipitates are higher at the interface zone. It is also interesting that the diameter of the BZO nanorods, 8–10 nm at the interface is approximately twice of the 5–7 nm size of the film within a pass. More importantly, distinct from the previous ~3 μm thick 15% Zr-added films, we do not observe big current-blocking second phase particles and misoriented grains in this sample from the TEM images^35^. To confirm this finding, we conducted large area back-scattered electron imaging on this 20% Zr-added, ~3.2 μm thick REBCO film. Figure 2(d) is a part of the image cropped from a wider image scanned along the sample width. All cracks and scratches in the image are from sample polishing. The image clearly shows the homogeneous microstructure along the width and the absence of large misoriented grains and big second phase particles; only occasional ~1 μm small second phase particles are observed.

**Figure 3:**
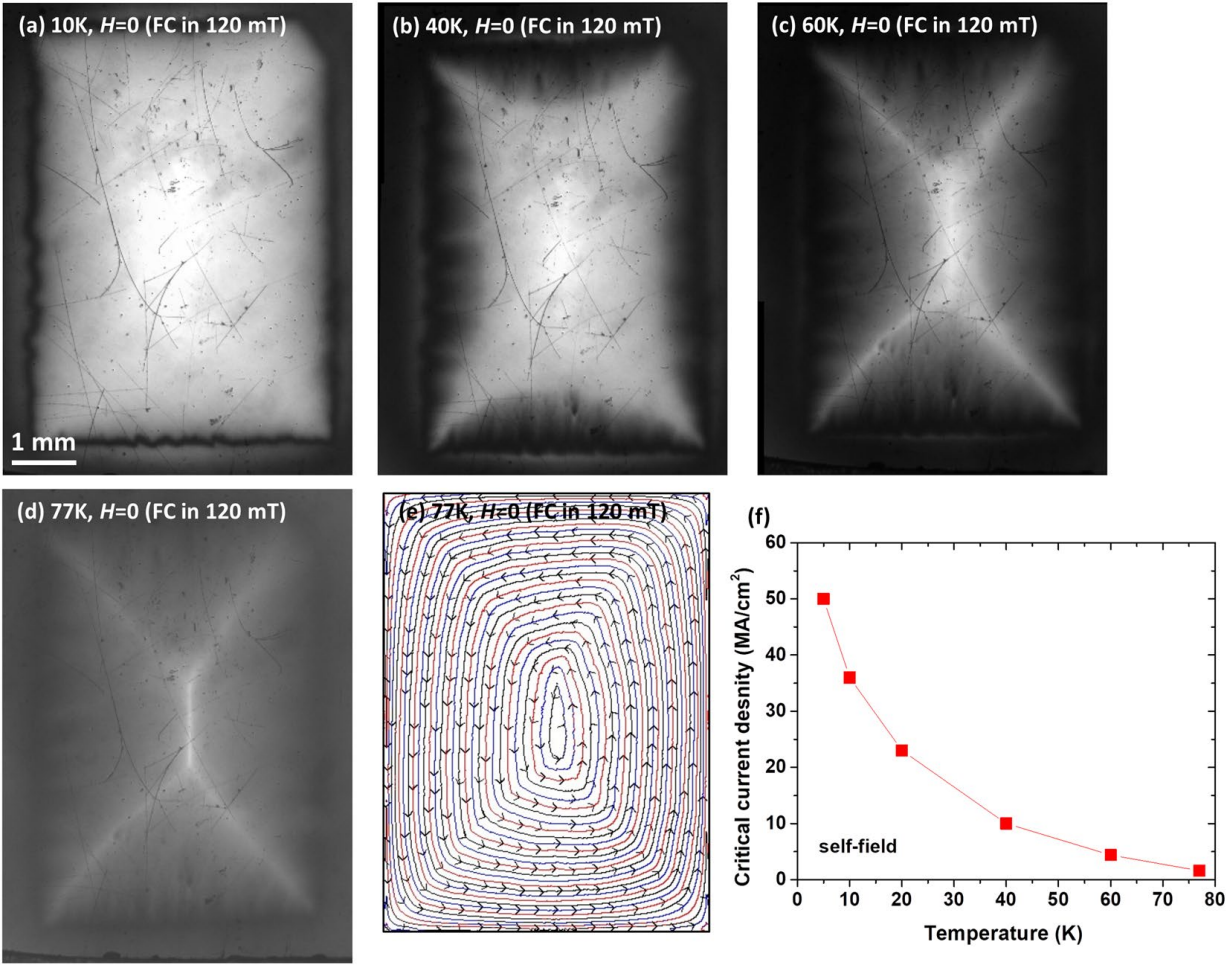
Magneto-optical microscope of the local trapped field after the sample cooled from room temperature in a 120 mT background field to (**a**) 10 K, (**b**) 40 K, (**c**) 60 K, and (**d**) 77 K. (**e**) Current stream lines calculated for (**d**) that illustrates the uniform current distribution in the film. (**f**) is the calculated *J*_*c*_ from the penetrated magnetic field after zero-field cooling according to Bean model. (**a**) to (**e**), especially the “rooftop” pattern in (**d**) suggest the electromagnetic homogeneity of the film. For all images, the brighter areas correspond to high trapped magnetic field regions.

Magneto-optical imaging is a powerful tool to reveal the local electromagnetic homogeneity of REBCO coated conductors down to a ~10 μm scale^36^. Figure 3(a) to (d) present the magneto-optical images of local trapped magnetic field profile of this ~3.2 μm thick REBCO film after field cooling (FC) from room temperature in an external magnetic field of 120 mT applied perpendicular to the tape plane to temperatures ranging from 77 to 10 K. At 10 K in Fig. 3(a), the sample shows a uniform, fully trapped field. At a higher temperature of 40 K shown in Fig. 3(b), the sample shows a partially-trapped field due to the lowered *J*_*c*_. On further increasing the temperature up to 60 K, the trapped field tends to a “rooftop” pattern. Upon raising the temperature up to 77 K, the sample is in critical state and shows well developed “rooftop” pattern, as shown in Fig. 3(d). The calculated current stream lines for the 77 K field-cooled magneto-optical image in Fig. 3(d) are plotted in Fig. 3(e), showing very uniform current flow parallel to the perimeter of the tape. The magnetization *J*_*c*_ calculated from the flux penetration pattern under magnet field after zero-field-cooling (ZFC) is shown in Fig. 3(f). All above images verify the electromagnetic homogeneity of this ~3.2 μm thick film.

**Figure 4:**
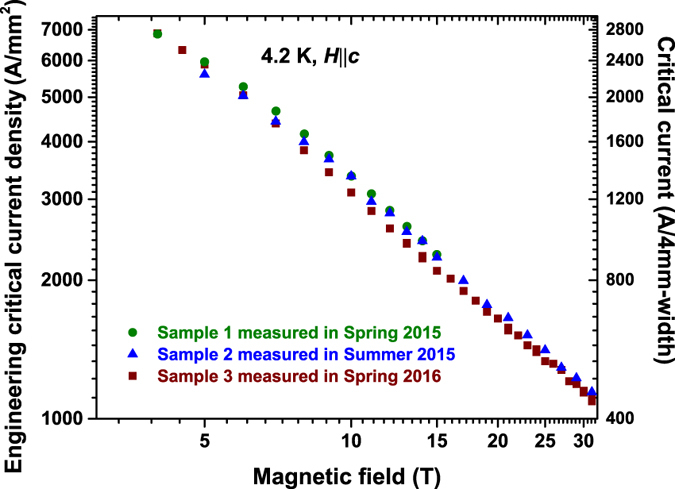
Field dependence of *I*_*c*_ normalized to 4 mm wide tape, and *J*_*e*_ at 4.2 K and magnetic fields up to 31.2 T. The ~3.2 μm thick film exhibits remarkable *J*_*e*_ > 1.0 kA/mm^2^ at magnetic fields up to 31.2 T. *J*_*e*_ was calculated based on the typical Hastelloy thickness of 50 μm and copper stabilizer thickness of 40 μm.

The field dependence of engineering current density *J*_*e*_ and critical current *I*_*c*_ at 4.2 K and field parallel to the *c*-axis from 2 up to 31.2 T are shown in Fig. 4. The critical current is normalized to that of a 4 mm wide tape. *I*_*c*_ at lower field is not shown because the critical current is above 0.9 kA of the power supply limit for the ~1 mm bridge. Three samples along the length of the original REBCO tape have been measured several times over a time period of one year. All three samples show almost same field dependence of *I*_*c*_, indicating the time stability and length homogeneity of *I*_*c*_ of this ~3.2 μm thick REBCO film along ~20 cm length. In the double logarithmic plot, similar to other REBCO coated conductors, a power-law field dependence, *I*_*c*_ ∝ *H* ^−*α*^ is observed, with *α* ≈ 0.89 between 10 and 31.2 T. *α* is widely used as a parameter for the strength of flux pinning^31^. The value shown by our ~3.2 µm thick film is lower than that of our 15% Zr-added ~0.9 μm thick film and higher than the ~3.0 μm 15% Zr-added sample^35^. The sample exhibits *I*_*c*_ above 1 kA at *H* up to 13 T, well above all other reported values^37^. Taking into account the ~50 μm thick substrate and ~40 μm thick surround copper stabilizer layer in commercial REBCO tapes, *J*_*e*_ of this 20% Zr-added ~3.2 μm thick REBCO film reaches 1 kA/mm^2^ at *H* up to 31.2 T, a real threshold for compelling, cost-effective applications.

## Discussion

*J*_*e*_ optimization depends on the specific background magnet field and temperature experienced in particular applications^1^. Thus, well-designed pinning defects are introduced into REBCO thick films for *J*_*e*_ enhancement via both *J*_*c*_ enhancement and thick REBCO film growth. High quality crystalline of REBCO growth on flexible substrates has been possible ever since the pioneer work of Iijima *et al*., to demonstrate biaxially-textured buffer layers by ion beam assisted deposition^38^. Very recently, an exceptional *I*_*c*_ over 1.5 kA/cm-width at 77 K and self-field was reported in a ~5 μm thick SmBCO film via a co-evaporation process^39^. Aiming for liquid nitrogen temperature and moderate field applications, Wee *et al*. reported a minimum *I*_*c*_ of 455 A/cm-width at 65 K and 3 T in a ~4 μm thick, 1 vol% BZO added PLD YBCO film^34^. Complementarily, for the purpose of very high field magnet applications at 4.2 K, in this work, we have reached record *J*_*e*_ over 1 kA/mm^2^ at 4.2 K and magnetic fields up to 31.2 T. Such high *J*_*e*_ can be partly attributed to the thicker REBCO layer with substantially reduced current blocking effects. Large second phase particles and misoriented grains observed in early MOCVD REBCO coated conductors not only substantially reduce the effective current-carrying area, but also deteriorated the BZO growth and the texture of REBCO films^35^. Because of the growth of large second phase particles and misoriented grains, our previous ~3 μm thick 15% Zr-added REBCO film showed *J*_*c*_ as low as the 7.5% Zr-added films, and 50% of the ~0.9 μm thick 15% Zr-added REBCO films^35^. Our current 20% Zr-added ~3.2 μm film is absent of such current blocking effects, supported by the XRD results, as well as the TEM/SEM observations, and verified by the “rooftop” magneto-optical patterns. Meanwhile, the high density of BZO nanorods well aligned along the *c*-axis also plays a key role for the high *J*_*e*_, as shown in the TEM images, and confirmed by the power-law field dependence of *J*_*c*_ with *α* ~ 0.89, higher than ~0.50 for the nominal pure REBCO films.

**Figure 5:**
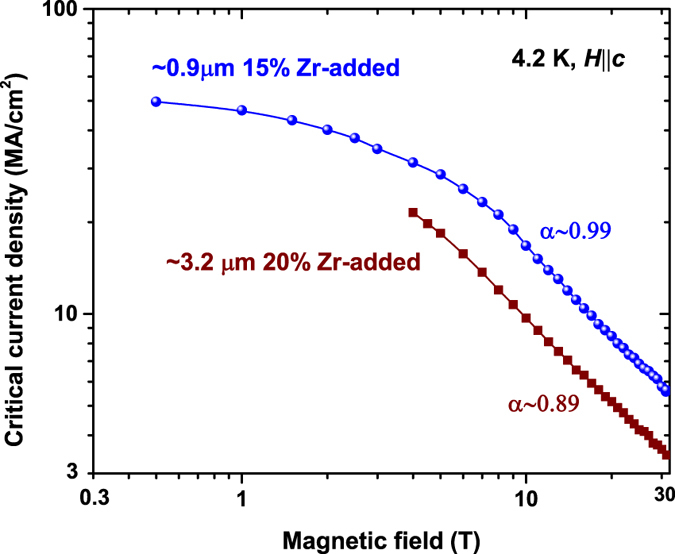
Comparison of *J*_*c*_(*H*||*c*) at 4.2 K and *H* up to 31.2 T of the ~3.2 μm thick, 20% Zr-added, and ~0.9 μm thick, 15% Zr-added REBCO film. The ~3.2 μm thick film shows not only lower *J*_*c*_ but also lower exponent for power-law field dependence of *J*_*c*_, implying reduced effective current-carrying area and degraded BZO nanorod growth in the ~3.2 μm film. Further increase in *J*_*e*_ is expected by overcoming of these two issues.

However, in comparison with the ~0.9 μm thick, 15% Zr-added film, *J*_*c*_ of this ~3.2 μm thick film is lower in the field regime from 2 to 31.2 T, as shown in Fig. 5. At 20 T, *J*_*c*_ ~ 5.2 MA/cm^2^ is about 38.8% lower than *J*_*c*_ ~ 8.5 MA/cm^2^ of the 15% Zr sample. This lower *J*_*c*_ can be partially ascribed to the lower weak pinning center density introduced by the BZO nanorods, supported by the lower *α* for the power-law dependence of *J*_*c*_, which is ~0.89 for the ~3.2 μm film, and ~0.99 for the ~0.9 μm 15% Zr-added film. In addition, the TEM image shows that the length of the BZO nanorod reduces with increasing REBCO layer thickness. Also, due to the multi-pass MOCVD process, a low density of thick and short BZO nanorods are observed in the 100–200 nm interface between two passes, as shown in Fig. 2(a) and (c). Further *J*_*e*_ improvement is expected via the growth optimization of BZO nanorods. The compelling high *J*_*e*_, stable and homogeneous *J*_*e*_ performance of this ~3.2 μm thick R&D film is appealing for high field magnet applications. In order for this result to be impactful, longer tape lengths have to be made with similar performance. Achieving consistent growth of aligned BZO nanocolumns in REBCO films with high levels of Zr addition in long tapes is a challenge with the conventional MOCVD process because of the need to control the tape temperature and precursor supply in a rather tight window. Such limitations can be overcome by our newly-developed Advanced MOCVD system, which offers homogeneous heating along the tape length, constant growth temperature during the long deposition for thick films and very stable precursor delivery. These features of the Advanced MOCVD system have enabled the growth of single-pass thick REBCO films above 4 μm showing *I*_*c*_(77 K, sf) ≈1.5 kA/12mm-width with equivalent *J*_*c*_ ≈ 3.0 MA/cm^2^ ^40^.

Further *J*_*e*_ improvement is crucial for REBCO cable development. *J*_*e*_ of our ~3.2 thick film exceeds the requirements, *J*_*e*_(4.2 K, 16 T) >1.5 kA/mm^2^ of the accelerator magnet for the future circular colliders^14^. However, from the perspective for magnet applications, multifilamentary round wire is strongly preferred for easy handling, isotropic performance and reduced ac loss. Several cabling techniques have been developed for REBCO coated conductors to produce suitable conductors for magnet builders with varying levels of success^41, 42^. The cabling unavoidably compromises the *J*_*e*_ advantage of REBCO coated conductors because of the need for mechanical support. We are working towards further improving the *J*_*e*_ of thick film Zr-added REBCO coated conductors to accommodate future magnet design and construction.

## Summary

We have demonstrated that, using a multi-pass MOCVD process, high *J*_*e*_ is feasible in thick Zr-added REBCO films above 3 μm. Such a high *J*_*e*_ has been possible through the elimination of large misoriented grains and suppression of big second-phase particles, as well as the growth of high density of *c*-axis self-assembled BZO nanorods. The extremely high *J*_*e*_(4.2 K, 31.2 T||*c*) above 1 kA/mm^2^ shown by this ~3.2 μm thick film exceeds the requirement of many very high field magnet applications at 4.2 K. Still, for REBCO cabling relevant for most magnet applications, further *J*_*e*_ improvement is pursued via further increasing REBCO layer thickness and controlling the pinning defect structure.

## Methods

### Sample preparation

The ~3.2 μm thick REBCO film was grown on a ~12 mm wide, ~50 μm thick standard buffered IBAD Hastelloy substrate using standard tetramethyl heptanedionate precursor by multi-pass MOCVD deposition^43^. Under the heater temperature of 960 °C, the tape was coated with a ∼1.1 μm thick REBCO film in the first pass, then cooled down to room temperature, and was coated with the second ∼1.1 μm thick film on the first layer in the second pass, and subsequently the third layer. After the third pass, a ∼2 μm thick silver layer was deposited on the REBCO film as a protection and current contact layer. In each pass, to introduce *c*-axis aligned BZO nanorods, 20 mol% Zr tetramethyl heptanedionate (thd) was added into the standard precursor solution with a nominal cation composition of (Gd_0.6_Y_0.6_)Ba_2.15_Cu_2.15_. The growth condition and the precursor are same for each pass.

### Measurements

The 4.2 K and high field four-probe critical current *I*_*c*_ measurements were performed on ∼1 × 10 mm bridges in a 52 mm warm bore 31 T Bitter magnet fitted with a 38 mm bore liquid He cryostat at the National High Magnetic Field Laboratory (NHMFL). The local current distribution was characterized by magneto-optical imaging on a sample with ~7 mm wide and ~8 mm long. To assess the current blocking effects of the potential second phase particles and the microstructure homogeneity, back-scattered electron imaging was conducted in a Zeiss 1540EsB scanning electron microscope (SEM). TEM was carried out in a JEOL ARM200cF to examine the size and distribution of the pinning defects. The phase identification and texture analysis were conducted using θ-2θ measurements in a Siemens D5000 x-ray powder diffractometer and an area detector in Bruker general area detector diffraction system. The thickness of REBCO layer, ∼3.2 μm was determined from the cross-section SEM images prepared by focused ion beam milling.
